# Block Copolymer–Sodium Oleate Complexes Through Electrostatic Interactions for Curcumin Encapsulation

**DOI:** 10.3390/ma18235375

**Published:** 2025-11-28

**Authors:** Evanthia Ganou, Michaila Akathi Pantelaiou, Varvara Chrysostomou, Karolina Olszowska, Barbara Trzebicka, Stergios Pispas

**Affiliations:** 1 Theoretical and Physical Chemistry Institute, National Hellenic Research Foundation, 48 Vassileos Constantinou Ave., 11635 Athens, Greece; euaganou@gmail.com (E.G.); akathi39@gmail.com (M.A.P.); chrysostomou.v@gmail.com (V.C.); 2 Department of Chemistry, National and Kapodistrian University of Athens, Panepistimiopolis Zografou, 15771 Athens, Greece; 3 Centre of Polymer and Carbon Materials, Polish Academy of Sciences, 34 ul. M. Curie-Skłodowskiej, 41-819 Zabrze, Poland; kolszowska@cmpw-pan.pl (K.O.); btrzebicka@cmpw-pan.pl (B.T.)

**Keywords:** polyelectrolyte complexes, block copolymers, sodium oleate, curcumin

## Abstract

Polyelectrolyte-based complexes have attracted attention, as the interaction of the oppositely charged components results in nanoparticle formation through an easy but highly efficient method, avoiding the use of strong solvents, extreme temperatures, and toxic chemicals. Sodium oleate (NaOL) is a widely used surfactant in the pharmaceutical industry due to its availability, eco-friendliness, and low cost. In the present study, the neutral-cationic block copolymer poly(oligo(ethylene glycol) methyl ether methacrylate)–b–quaternized poly(2-(dimethylamino) ethyl methacrylate) (POEGMA-b-Q(PDMAEMA)) is mixed with the anionic surfactant sodium oleate for the formation of nanoscale polyelectrolyte complexes through electrostatic interactions. Different weight ratios of copolymer to surfactant are studied. Then, the co-solvent protocol was implemented, and curcumin is successfully loaded in the formed particles for drug delivery applications. The size and morphology of the macromolecular complexes are examined via Dynamic Light Scattering (DLS) and Cryogenic Transmission Electron Microscopy (cryo-TEM). The methods that we have used have indicated that the polymer–surfactant complexes form spherical complexes, worm-like and vesicle-like structures. When curcumin was introduced, encapsulation was effectively achieved into micelles, giving rise to vesicle-like shapes. The success of curcumin encapsulation is confirmed by Ultraviolet–Visible absorption (UV–Vis) and fluorescence (FS) spectroscopy. POEGMA-b-Q(PDMAEMA)–sodium oleate polyelectrolyte complexes revealed promising attributes as efficient drug carrier systems for pharmaceutical formulations.

## 1. Introduction

Block copolymers are macromolecules that present the properties of their respective constituents in their separate intramolecular regions (blocks) [1]. A vast array of scalable techniques allow the formation of such polymers, often with a high degree of precision in molecular weight distribution [2]. As a result, macromolecules that exhibit an endless variety of block combinations can be produced suitable for corresponding applications [3,4] such as biomedicine [5]. One of the most common examples are copolymers that consist of blocks with different solubility in certain solvents [6]. Poly(oligo(ethylene glycol) methyl ether methacrylate) (POEGMA) has attracted considerable attention due to its exceptional properties [7]. It is a non-toxic and thermosensitive polymer suitable for biomedical applications [8,9,10]. Quaternized poly(2-(dimethylamino) ethyl methacrylate) (Q(PDMAEMA)) is a cationic, biocompatible polymer widely used in biomedical applications, such as antibacterial patches [11], insulin nanocarriers [12], and biocidal coatings [13,14].

Copolymers that combine hydrophobic and hydrophilic blocks are called amphiphilic block copolymers. This category of copolymers has been used extensively on surface coatings [15], semi-crystalline materials [16], and membrane separation [17]. Amphiphilic polymers can self-assemble in water and encapsulate substances that are water-insoluble. This is achieved via the formation of vehicles with a hydrophobic core, thus rendering such macromolecules highly significant for drug delivery applications. In a previous study by Chroni et al. [18], the hydrophobic drug losartan potassium (LSR) was shown to be encapsulated in the hydrophobic region of the nanostructures formed by an amphiphilic block copolymer, poly(n-butyl acrylate)-block-poly(oligo(ethylene glycol) methyl ether acrylate) (PnBA-b-POEGA).

Sodium oleate is an anionic surfactant that consists of an oleate anion that forms an ionic bond to a sodium cation [19]. It is environmentally friendly [20], easily accessible, and affordable [21]. Sodium oleate can bind with positively charged polymers due to electrostatic interactions between them [22]. The biocompatibility of sodium oleate, along with the ability to interact with polymers, make its use promising for biomedical applications [23].

Polyelectrolytes (PEs) are a category of ionically charged polymers that are either found in nature or synthesized in a lab [24]. When positively or negatively charged polyelectrolytes are combined in aqueous media with charged substances, they assemble into aggregates through electrostatic interactions. These aggregates are called polyelectrolyte complexes (PECs) [25]. A common method for forming PECs is through the combination of a PE and an ionic surfactant, like sodium oleate [26]. The addition of surfactants leads to the fabrication of micelles of larger diameter than those consisting of pure amphiphilic polymers. The larger micelles are capable of encapsulating substances efficiently [27]. Interest has risen for PEC applications in drug delivery systems, due to their capacity to enclose substances of low molecular weight like drug molecules, as well as larger molecules (proteins, nucleic acids, and peptides) [28]. This effect proves useful to the development of more patient-friendly drug delivery nanocarriers [29]. Kizhakkedathu et al. combined cationic copolymers and sodium oleate, creating soluble spherical nanoparticles, verified through pyrene fluorescence studies, that consist of a hydrophobic core surrounded by a hydrophilic corona [30]. The authors of the aforementioned work suggested that the produced micelles would be able to encapsulate hydrophobic drug molecules. Another challenge in modern pharmacology is the low bioavailability of drug molecules like docetaxel, a compound used in chemotherapy [31]. To this end, Chen et al. used poly(lactic-co-glycolic acid)-b-poly(ethylene glycol), PLGA-b-PEG, and sodium oleate to fabricate spherical drug carriers of docetaxel through a solvent diffusion protocol [32]. In this case components were co-assembled through hydrophobic interactions. In vitro studies using cells from a cancer cell line indicated nanoparticle absorption through cellular uptake pathways, resulting in greatly increased bioavailability and targeted drug delivery.

The curcumin molecule is a polyphenol obtained from the turmeric plant, *Curcuma longa* [33]. It is typically used as a food additive, food coloring, and in cosmetics. Furthermore, the molecule attracts great interest in anti-cancer research due to its iron-chelating activity [34], activation of cell apoptosis pathways [35], and ability to regulate cell signaling pathways [36] and to interact with several immune mediators [37]. Factors that hinder the use of curcumin in anti-cancer research are its insolubility in water, lack of selectivity, and fast metabolism from the human body [38]. To counteract these issues and ensure efficient drug delivery, the option of encapsulating curcumin in polymeric nanocarriers is examined. In our group’s previous research, by Pantelaiou et al., poly(oligoethylene glycol methyl ether methacrylate-co-methyl methacrylate), P(OEGMA-co-MMA), an amphiphilic statistical copolymer was synthesized via reversible addition–fragmentation chain-transfer polymerization (RAFT) to form nanocarriers of curcumin in aqueous media [39]. The hydrophobic core of those carriers provided a suitable environment for curcumin encapsulation, while the hydrophilic exterior provided good solubility in water and biocompatibility. The results indicated nanoparticle stability, as well as high encapsulation efficiency. Also, star-like structures with poly(hydroxy propyl methacrylate) (PHPMA) cores and poly(oligoethylene glycol methacrylate) (POEGMA) synthesized by RAFT were studied as carriers for curcumin by Sentoukas [40]. A small number of studies has been conducted on copolymer polyelectrolyte systems that contain both polymers and surfactant for the encapsulation of hydrophobic drugs. As such, systems that are formed through the interplay of both ionic and hydrophobic interactions have not been studied sufficiently. Furthermore, the surfactant used is biocompatible and has not been utilized in similar studies with copolymers. While a great number of studies use PEG for its biocompatibility, we have selected an alternative copolymer that includes POEGMA block. Like PEG, this polymer is also biocompatible but more versatile and promising for added functionality [7]. Moreover, the copolymer POEGMA-b-Q(PDMAEMA) exhibits a positive charge within the polyelectrolyte part and can also be utilized for DNA transport research.

In this study, we opted to form polyelectrolyte-based complexes as candidates for encapsulation of curcumin. To this end, poly(oligo(ethylene glycol) methyl ether methacrylate)–b–quaternized poly(2-(dimethylamino) ethyl methacrylate) (POEGMA-b-Q(PDMAEMA)) was synthesized in-house via RAFT polymerization. Mixing of aqueous solutions of block copolymer and surfactant leads to the formation of POEGMA-b-Q(PDMAEMA)) and sodium oleate complexes via electrostatic interactions. The charge of the resulting nanoparticles (P:S) as well as their building blocks was determined via zeta potential measurements. FS was used to determine the hydrophilicity of the nanoparticles and individual macromolecules. DLS was used to ascertain the size and size distribution of nanoparticles formed by block copolymer–surfactant complexation. The resulting nanoparticles were then tested for stability over the span of a week after preparation, showing good size retention. The relation of nanoparticle size to the salinity of the aqueous medium was tested for NaCl concentrations ranging from 0 to 0.5 M. Nanoparticles with encapsulated curcumin (P:S:C) were formed via a co-solvent protocol. DLS measurements on the loaded nanocapsules indicated a general increase in radius in every sample produced. Stability testing for the loaded nanoparticles was also conducted 14 days after preparation. The geometry of unloaded and loaded nanoparticles was also tested via cryo-TEM. The encapsulation efficiency and loading of curcumin within the nanoparticles were confirmed via UV–Vis and FS measurements.

## 2. Materials and Methods

### 2.1. Materials

**Figure 1:**
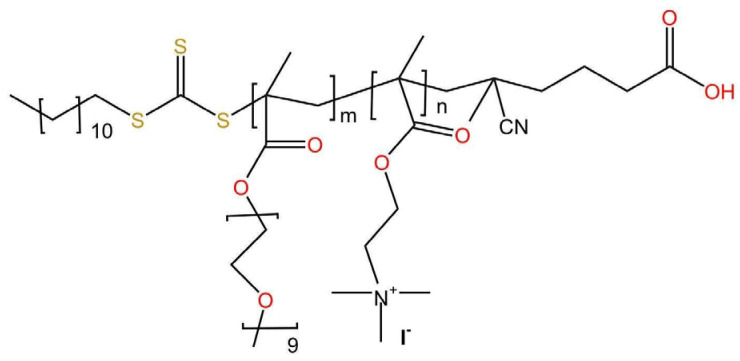
Chemical structure of POEGMA-b-Q(PDMAEMA).

In this study, nanocarriers were produced by using the in-house synthesized double hydrophilic POEGMA-b-Q(PDMAEMA) block copolymer (M_w_ = 19,700 gmol^−1^, Đ = 1.28, 47 wt% POEGMA and 53 wt% Q(PDMAEMA)) where OEGMA with nine ethylene glycol units was utilized. The number of monomers in POEGMA and PDMAEMA is 18 and 34 units, respectively. The chemical structure of POEGMA-b-Q(PDMAEMA) is depicted in Figure 1. The synthesis and the quaternization of copolymer POEGMA-b-PDMAEMA was achieved as described in the work by Haladjova et al. [41]. Briefly, the copolymer was dissolved in tetrahydrofuran at a 2% *w*/*v* ratio. Afterwards, 0.95 mmol of methyl iodide was added and reacted with POEGMA-b-PDMAEMA for 24 h at room temperature. As indicated in the relevant work, the reaction is almost quantitative and ^1^H NMR analysis demonstrated above 97% conversion of the tertiary to quaternary amine. The degree of polymerization for POEGMA is calculated to be 64 and for Q(PDMAEMA) is calculated to be 35. Ethanol (EtOH), pyrene, sodium oleate (NaOL), and curcumin (CUR) were obtained from Sigma-Aldrich (Burlington, MA, USA).

### 2.2. Co-Assembly of Polyelectrolyte–Surfactant

The co-assembly of the polymer and surfactant was undertaken in deionized water. The concentrations of the solutions were C = 1 × 10^−3^ g/mL. Initially, the copolymer and the surfactant were dissolved in deionized water. The neat polymer solution and neat sodium oleate solution were examined as reference. Following that, in every sample, the required volume of sodium oleate was injected into 5 mL of the polymer solution under fast stirring conditions for 10 min. The final volume of the samples was adjusted to 10 mL with the addition of deionized water. The solutions were kept on the bench at room temperature overnight. Four samples with different mass ratios of surfactant relative to copolymer were prepared, P:S 20%, P:S 40%, P:S 60%, and P:S 80%, with their respective charge ratios (negative/positive charge) being 0.37, 0.76, 1.14, and 1.52.

### 2.3. Curcumin Encapsulation in Polyelectrolyte Complexes

The co-solvent protocol was used to encapsulate the hydrophobic molecule curcumin into the polymer–surfactant complex. The types of complexes selected for curcumin loading were P:S 40% and P:S 60%. The concentration of curcumin loaded was maintained in each sample equal to C_cur_ = 0.5 mg/mL. For the dissolution of both components of polymer–surfactant mixture and curcumin, ethanol was chosen due to its volatility and polarity. The ethanol was evaporated overnight to ensure that any quantity that remained within the solution is in the ppm range. Firstly, curcumin was dissolved in ethanol and left for 30 min on the bench, at room temperature. The polymer was added in ethanol and left overnight until dissolution. The same process was followed for sodium oleate that was added into the previously prepared curcumin–ethanol solution. Then, the solutions of polymer and surfactant–curcumin were combined. The three-component solution that was formed was injected in 10 mL of deionized water and kept for two hours at 60 °C under stirring, for ethanol evaporation. The mixed solutions were kept overnight at room temperature and under stirring. Prior to measurements, the final solution volume was increased to 10 mL via addition of water.

### 2.4. Impact of Ionic Strength and Fetal Bovine Serum (FBS)

The effect of ionic strength on the developed polyelectrolyte–surfactant complex was investigated via salt addition. An initial solution of NaCl 1 M was created. From that solution, appropriate volumes were pipetted to 1 mL of the tested nanoparticle solution to ensure a gradually increasing concentration from 0.05 mg/mL to 0.5 mg/mL of salt. The FBS:PBS solution (1:9 *v*/*v*) was added in each curcumin-loaded POEGMA-b-Q(PDMAEMA)/NaOL sample for examination of the impact of FBS in the nanoparticles. In particular, 1.5 mL of FBS:PBS was used with 150 μL of the tested nanoparticle solution, allowed to sit for an hour, and then analyzed using the DLS instrument. FBS and PBS were provided by Sigma-Aldrich.

### 2.5. Characterization of Nanocomplexes

#### 2.5.1. Dynamic Light Scattering (DLS)

DLS measurements were conducted with an ALV/CGS-3 compact goniometer system (ALV GmbH, Hessen, Germany). A JDS Uniphase 22 mW He-Ne laser source (632.8 nm wavelength, JDS, Milpitas, CA, USA), an ALV/LSE-5003 control unit (ALV GmbH, Hessen, Germany) serving as the electronic interface, and a multi-τ correlator with 288 channels (ALV5000/EPP, ALV GmbH, Hessen, Germany) make up the DLS system utilized. Prior to the analysis, 0.45 μm PVDF filters were used for filtration of all solutions. The autocorrelation functions and the scattered light intensity were calculated from the mean value of five measurements at a scattering angle of 90° for each sample solution. The cumulants method and the CONTIN algorithm in the manufacturer’s software were used for the analysis of the DLS data, resulting in hydrodynamic radius referred to as R_h,cum_ and R_h,cont_, respectively. The DLS analysis also determined the scattering intensity (I_90_) and the polydispersity index (PDI) of the sample.

#### 2.5.2. Electrophoretic Light Scattering (ELS)

ELS measurements were performed in order to determine the surface charge of the particles in solution. The device employed was the Nano Zeta Sizer (Malvern Panalytical, Malvern, UK) from Malvern. At a scattering angle of 173°, analysis was conducted using a 4 mW He–Ne laser (633 nm wavelength) as the light source (Malvern Panalytical, Malvern, UK). The Smoluchowski equation was applied for the data analysis. After 20 scans of each sample, the average value was calculated.

#### 2.5.3. Cryogenic Transmission Electron Microscopy (Cryo-TEM)

Cryogenic Transmission Electron Microscopy (cryo-TEM) images were obtained using a Tecnai F20 X TWIN microscope (FEI Company, Hillsboro, OR, USA) equipped with a field emission gun and operating at an acceleration voltage of 200 kV. Images were recorded on the Gatan Rio 16 CMOS 4K camera (Gatan Inc., Pleasanton, CA, USA) and processed with Gatan Microscopy Suite (GMS) software version 3.31.2360.0 (Gatan Inc., Pleasanton, CA, USA). Specimen preparation was performed by vitrification of the aqueous solutions on grids with holey carbon film (Quantifoil R 2/2; Quantifoil Micro Tools GmbH, Großlöbichau, Germany). Prior to use, the grids were activated for 15 s in oxygen plasma using a Femto plasma cleaner (Diener Electronic, Ebhausen, Germany). Cryo-samples were prepared by applying a droplet (3 μL) of the suspension to the grid, blotting with filter paper, and immediately vitrifying in liquid ethane using the fully automated blotting device Vitrobot Mark IV (Thermo Fisher Scientific, Waltham, MA, USA). After preparation, the vitrified specimens were kept under liquid nitrogen until they were inserted into the cryo-TEM holder Gatan 626 (Gatan Inc., Pleasanton, CA, USA) and analyzed in the TEM at −178 °C.

#### 2.5.4. Fluorescence Spectroscopy (FS)

FS analysis identified the hydrophobicity of the polyelectrolyte complexes via pyrene encapsulation. Furthermore, curcumin-loaded nanocarriers’ fluorescence was measured. Each sample was diluted at a 1:10 ratio with deionized water. A NanoLog Fluorometer (Horiba Jobin Yvon, Kyoto, Japan) was used for the measurements. The excitation source was a NanoLED 440 nm laser with 100 ps pulse width (Horiba Jobin Yvon, Kyoto, Japan). UV TBX-PMT (250–850 nm) by Horiba Jobin Yvon (Kyoto, Japan) was the detector for the measurements.

#### 2.5.5. Ultraviolet–Visible Absorption Spectroscopy (UV–Vis)

Samples were diluted at a ratio of 1:10 with deionized water and placed in quartz cuvettes. The Perkin–Elmer (Lambda 19, Waltham, MA, USA) UV–Vis–NIR spectrophotometer was used for absorption spectroscopy measurements.

## 3. Results

### 3.1. POEGMA-b-Q(PDMAEMA)–Sodium Oleate Complexation

#### 3.1.1. POEGMA-b-Q(PDMAEMA) Characteristics

The synthesis of the block copolymer POEGMA-b-Q(PDMAEMA) with molecular weight (M_W_) 19,700 gmol^−1^ was conducted in-house as described by Haladjova et al. [41] using RAFT polymerization. It was demonstrated by earlier studies [42] that the resulting copolymer is able to form electrostatically assembled complexes with anionic polyelectrolytes in water. Neutral POEGMA blocks provide the hydrophilic properties of the copolymer, enabling its solubility in an aqueous environment. Q(PDMAEMA) contains cationic charges where an anionic surfactant can bind. The interaction of block copolymer with sodium oleate results in a micellar-like polyelectrolyte complex, with a hydrophobic core formed by the NaOL hydrophobic tails bound to Q(PDMAEMA) blocks and hydrophilic coronas of POEGMA blocks, which can encapsulate active hydrophobic species.

#### 3.1.2. POEGMA-b-Q(PDMAEMA) and Sodium Oleate Interactions

The complexation of surfactant and Q(PDMAEMA) is caused by electrostatic interactions between the negative and positive functional groups present in the corresponding molecules. In the present study the complexes were formed in an aqueous environment as described in Section 2.2, wherein the weight ratio between block copolymer and surfactant was varied to examine the influence of the sodium oleate content on the physicochemical characteristics of the mixed system. The resulting ratios of Polymer/Surfactant (P:S) were 20%, 40%, 60%, and 80% (*w*/*w*). The percentage parameter indicates the mass ratio of surfactant to copolymer, represented as the mass of surfactant per mass of copolymer.

**Figure 2:**
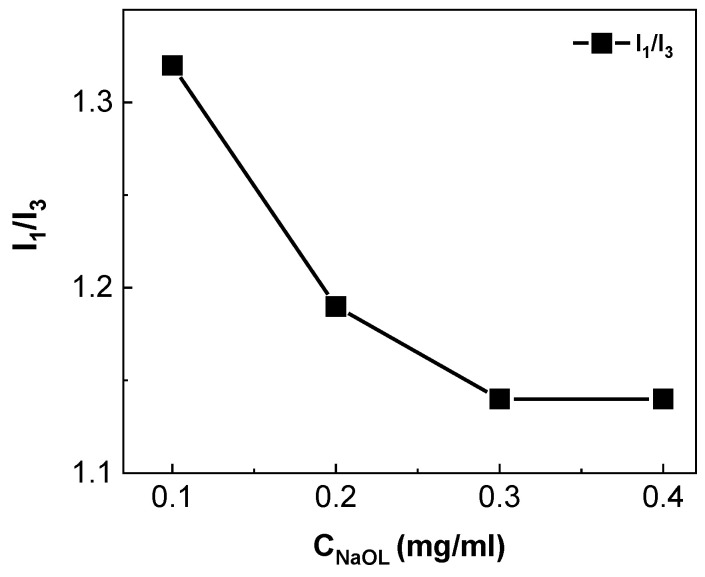
I_1_/I_3_ ratio versus the concentration of sodium oleate in the complexes.

The complexes resulting from four different ratios were examined for their size, charge, and hydrophilicity, via DLS, zeta potential, and FS, respectively. The relevant results appear in Table 1. Results from corresponding measurements on the pure copolymer solution are denoted by P, pure sodium oleate solution by S, and polymer–sodium oleate solutions by P:S followed by their ratio. Regarding P, DLS results showed two values for R_h_, one at R_h_ = 8.5 nm and a larger one at R_h_ = 84 nm, and a relatively low scattering intensity of 430 Κcps when compared to all other samples. The large R_h_ values most likely correspond to large aggregates and the smaller ones to free copolymer chains. Conversely, measurements on sodium oleate solution indicated the highest scattering intensity of all samples and hydrodynamic radius at R_h_ = 39 and R_h_ = 172 nm. The gradual increase in sodium oleate in the complexes is followed by an increase in R_h_ and scattering intensity. In P:S 40% and P:S 60% samples, two peaks are visible, one that corresponds to smaller and one that corresponds to larger aggregates, with the scattering intensity of the former aggregate populations being higher than the latter ones. Zeta potential measurements were conducted to estimate the charge of copolymer and sodium oleate species in solution. The decrease in zeta potential values agrees qualitatively with the increase in charge ratio mentioned in paragraph 2.2. Namely, the P:S 20% sample with the positive zeta potential value has a charge ratio calculated well below 1.0. Concerning P:S 60% and P:S 80%, the negative zeta potential values stem from the excess negative charge of NaOL molecules at this composition. In Table 1 a value of 42 mV from measurements on the copolymer points to a positive charge, whereas corresponding measurements on sodium oleate solutions produced a value of −71 mV indicating a negative charge. As is apparent in Table 1, increasing the sodium oleate content in the complexes led to the decrease in zeta potential. The complex with the lowest value was the one with the highest percentage of sodium oleate (P:S 80%). The hydrophilicity of the copolymer, sodium oleate, and copolymer/sodium oleate complexes was examined via fluorescence spectroscopy, with the I_1_/I_3_ ratio of pyrene probe molecules indicating the polarity of each system. Pyrene was added in the complexes as a fluorescent probe due to its ability to be encapsulated within the hydrophobic areas of aggregates [30]. Figure 2 shows the intensity ratio of the first and third peak (I_1_/I_3_) in FS spectra produced by measurements on the corresponding P:S complexes for increasing sodium oleate content. Higher values of I_1_/I_3_ indicate a more hydrophilic character of the corresponding complex. In Table 1 POEGMA-b-Q(PDMAEMA) is shown to exhibit a relatively high I_1_/I_3_ value. This indicates a more pronounced hydrophilic character for the copolymer in aqueous media, whereas values of I_1_/I_3_ below 1 for sodium oleate indicate high hydrophobicity of the surfactant aggregates in water solutions. As far as the polyelectrolyte complexes are concerned, the value of the I_1_/I_3_ ratio gradually decreases with the increase in surfactant, indicating the presence of hydrophobic areas.

Figure 2 demonstrates the decrease in the value of I_1_/I_3_ ratio for samples of complexes with the increase in the concentration of the surfactant. The four complexes show considerable hydrophobicity (I_1_/I_3_ < 1.32) and the lowest attainable value of 1.14 indicates a plateau, meaning that no further alteration will appear in the internal polarity of the nanosystems with further addition of sodium oleate.

**Figure 3:**
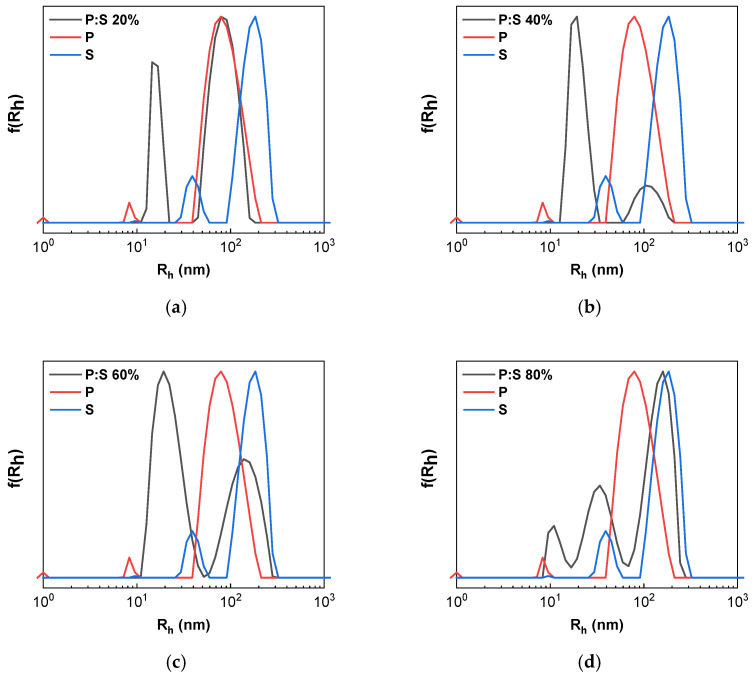
Size distributions from Contin analysis of DLS measurements: (**a**) P:S 20%; (**b**) P:S 40%; (**c**) P:S 60%; (**d**) P:S 80% where (black) is the complex compared to pure copolymer (red) and pure surfactant (blue) in aqueous solution.

Figure 3a–d show the DLS size measurements of the four different complexes obtained. The sample with the smallest percentage of sodium oleate produces a peak that is nearly identical to that produced by the pure copolymer sample. P:S 20% also produces the lowest values for R_h_ compared to the rest of the complexes, implying copolymer–NaOL interactions that result in smaller aggregates. In Figure 3a, the polymer (red) and the complex (black) curves for P:S 20% almost coincide, though in the pure surfactant curve (blue), the peak that corresponds to the complex is not visible, meaning a complete complexation of surfactant and polymer has occurred. Furthermore, another peak in the complex curve appears at 19 nm, indicating a population of smaller complexes. On the other hand, the sample with the highest NaOL percentage presents similar behavior to pure sodium oleate solutions. The P:S 40% curve (Figure 3b) indicates a greater nanocomplex population with a R_h_ of 20 nm. The peak height at 60 nm is significantly decreased, meaning that the complex causes a greater degree of scattering than the scattering from remaining pure polymer. It is possible that any surfactant micelles have de-aggregated. P:S 60% also produces a peak at 20 nm for R_h_ value. Moreover, the second large-size peak is shifted to 140 nm, probably indicating the formation of larger surfactant micelles which scatter light more intensively.

The P:S 80% sample presents the maximum excess of surfactant resulting in large scattering of light from NaOL micelles. The peak of the P:S 80% sample coincides with the one on the surfactant curve, whereas the 20 nm sized aggregate peak is significantly less visible. For P:S 80% complexes, the highest polydispersity index is evident. There are three peaks, indicating different nanomaterial size populations of complexes formed by the copolymer and surfactant. Electrostatic interactions and complex formation are observed in every sample. The peaks at smaller R_h_ values indicate the free polymer chains present within the P:S 80% solution.

The solutions of the complexes were also examined for their stability. Thus, DLS measurements were conducted one week after the preparation of the initial solutions. All samples exhibited no discernible precipitation. The corresponding DLS results are shown in Table 2. When compared to the ones produced one day after the preparation of the samples (Table 1), it is shown that the peaks corresponding to P:S 20% remain centered on the same values as before. However, the peak at 15 nm is decreased when measured a week after preparation. For P:S 40% the peak center at the smaller corresponding R_h_ value has remained relatively constant, although it appears greatly broadened. Moreover, all samples exhibit a decreased scattering intensity. The scattering intensity is altered due to the self-assembly process of the components. The more stable samples were P:S 20% and P:S 40% due to the excess of copolymer and the stabilizing effect of hydrophilic POEGMA blocks. The P:S 60% and P:S 80% were less stable because of the excess of surfactant, creating micelles which may also interact with the copolymer chains as individual entities, causing either sedimentation or exchange of molecules and rearrangement of the aggregates in the mixed aqueous solutions. As sedimentation is not observed macroscopically, possibly due to low values of concentration, such a mechanism is currently hypothetical.

**Figure 4:**
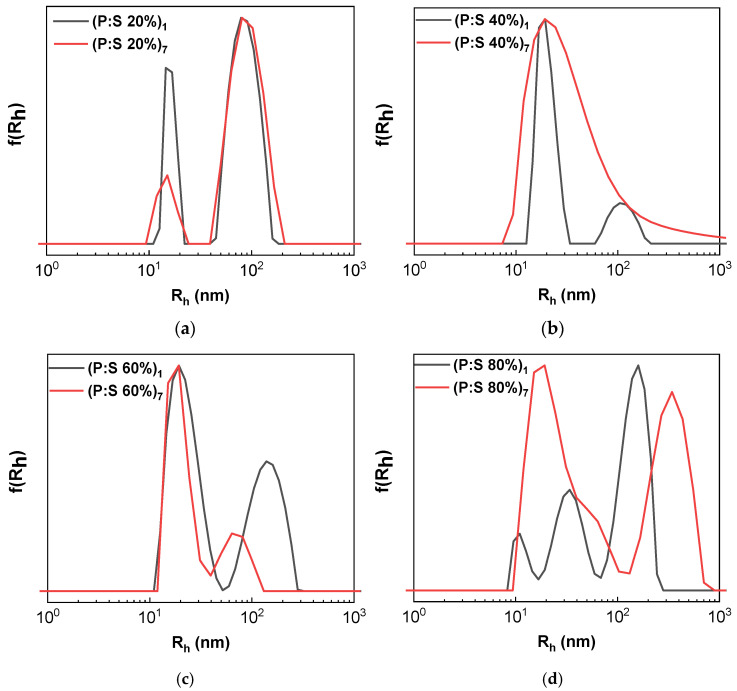
Size distributions from DLS one day (black) and one week after preparation (red) of the copolymer–NaOL complexes: (**a**) P:S 20% stability testing; (**b**) P:S 40% stability testing; (**c**) P:S 60% stability testing; (**d**) P:S 80% stability testing.

Figure 4a–d contain the DLS curves from the samples one day after and one week after preparation. The P:S 40% has a broadened peak thus an altered structure due to the passage of time in this sample. The P:S 60% sample has similar behavior to the initial sample. P:S 80% appears with larger particles.

#### 3.1.3. Addition of Sodium Chloride in P:S 40% and P:S 80% Solutions of Complexes

**Figure 5:**
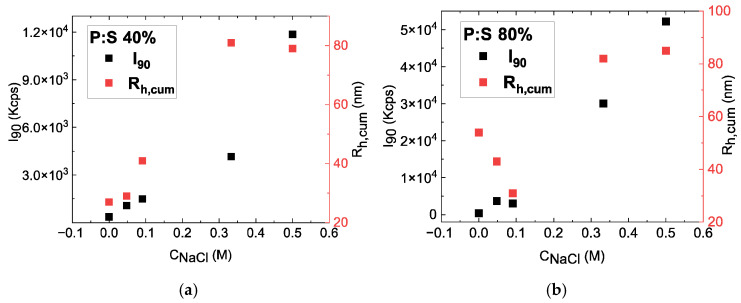
Intensity and R_h,cum_ by cumulant analysis of DLS data as a function of NaCl concentration for (**a**) P:S 40% complexes and (**b**) P:S 80% complexes.

The ionic strength of solutions in water is significant for applications in biology, medicine, and healthcare. Electrostatic interactions between the block copolymer and sodium oleate may be altered due to the presence of NaCl, and formation of nanoaggregates could be hindered. For this reason, the physicochemical characteristics of P:S 40% and P:S 80% complexes were examined via DLS under conditions of variable salinity. The samples tested were first checked for stability and then NaCl was added. In Figure 5 the scattering intensity, I_90_ (black) and R_h_ (red), are plotted as a function of NaCl concentration in the aqueous solution. In Figure 5a an increase in scattering intensity that is accompanied by an increase in R_h_ is observed for increasing NaCl concentration in P:S 40%-containing samples, excluding a small decrease in R_h_ at 0.5 M, whereas in Figure 5b, P:S 80% exhibits a decreasing R_h_ between 0 and 0.1 M of NaCl concentration which then shows a major increase above 0.3 M of NaCl, demonstrating a more complex behavior of aggregate radius in relation to salinity. This complex behavior can be attributed to the larger percentage of sodium oleate within the complex. The scattering intensity of P:S 80%, however, exhibits similar behavior to that of P:S 40%, as it increases with the addition of NaCl. The overall increase in scattered intensity and R_h_ indicates an increase in the mass and size of the complexes, probably due to secondary aggregation of the initially formed complexes as a result of screening electrostatic interactions with the presence of excess ions in the systems.

#### 3.1.4. Cryo-TEM Imaging on P:S 40% and P:S 60% Complexes

**Figure 6:**
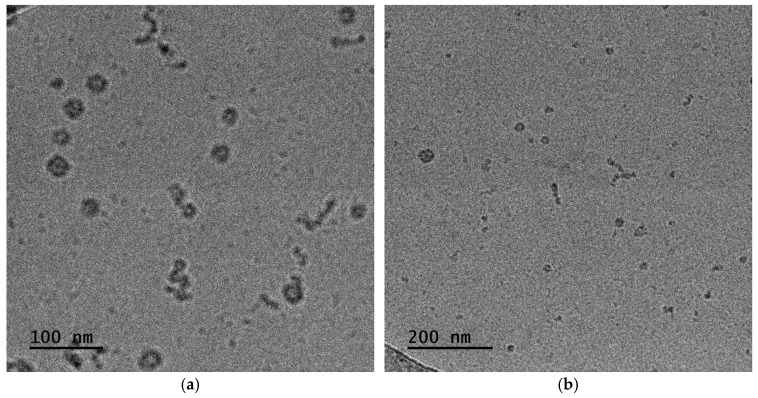
Cryo-TEM images for solutions of copolymer–NaOL complexes: (**a**) P:S 40%; (**b**) P:S 60%.

Cryo-TEM is widely used for imaging and examining the structural characteristics of nanoparticles. DLS analysis of P:S 40% in Section 3.1.2 demonstrated that the greater population corresponds to R_h_ around 20 nm. Figure 6a contains an image produced by cryo-TEM of the above-mentioned sample which displays particles of comparable diameters. Regarding P:S 60% shown in Figure 6b, the micelle core diameter indicates an average diameter of 35 to 40 nm with the maximum reaching 60 nm. The larger population in DLS corresponds to R_h_ at 19 nm, while a smaller one is at 139 nm. The reduced population may not be quantifiable from this image since TEM produces a snapshot of a part of the solution and not the whole sample. Due to insufficient contrast, the corona of the micelles cannot be seen using cryo-TEM. The DLS measures the whole volume of the complex and hence displays a higher R_h_ value equal to 65 nm.

**Figure 7:**
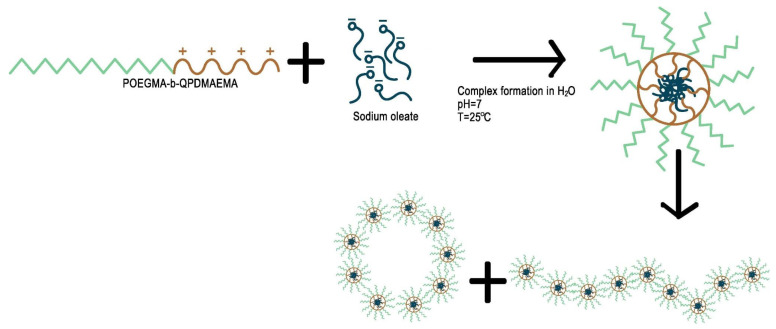
Schematic illustration of the POEGMA-b-QPDMAEMA/sodium oleate complexation and structure formation in aqueous solution.

The assembly of surfactant and polymer complexes in water is expected to be like the graphical illustration in Figure 7. The expectation aligns with the picture of the cryo-TEM where both spherical and worm-like structures are depicted. Worm-like structures seem to be composed of smaller and denser spherical structures (probably primary copolymer–NaOL complexes of core–shell micellar morphology, Figure 6a). Additionally, larger spherical structures in Figure 6a resemble small vesicles (hollow structures with an outer membrane with larger contrast). Such vesicles may be a result of structural transformations of worm-like structures. In any case the final morphology is expected to be dictated by space requirements due to steric hindrance for the long oleate hydrophobic chains after complexation with the copolymer chains. Structures seem to be similar in the case of P:S 60% complexes despite the increase in NaOL content which could lead to greater variability of the observed co-assembled structures. The structures formed are primarily small vesicles and several worm-like structures (Figure 6b).

### 3.2. Block Polyelectrolyte–NaOL Complexes as Curcumin Nanocarriers

#### 3.2.1. Encapsulation of Curcumin in Copolymer–Surfactant Complexes

**Figure 8:**
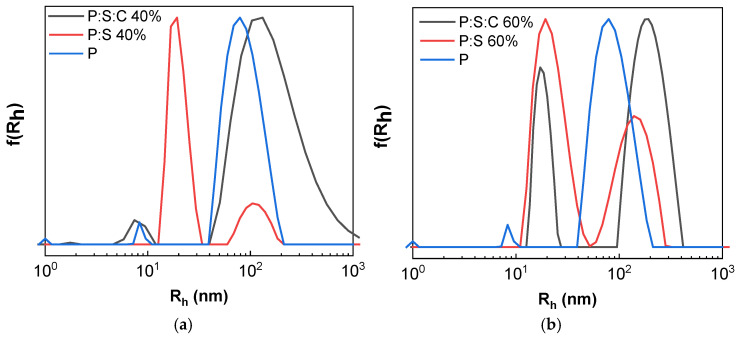
Size distributions from DLS for (**a**) P:S:C 40% (black) compared to P:S 40% (red) and plain polymer (blue) and (**b**) P:S:C 60% (black) compared to P:S 60% (red) and plain polymer (blue).

Curcumin was utilized for encapsulation into the polyelectrolyte complexes. Curcumin is hydrophobic and encapsulation can be achieved within the aggregates in aqueous media [43]. For the preparation of the polymer/sodium oleate/curcumin-loaded nanocarriers, the co-solvent protocol described in the Methodology Section was followed by using ethanol as a common solvent. The complexes used for encapsulants are P:S 40% and P:S 60%. The following naming convention was used: Polymer/Surfactant/Curcumin (P:S:C) with the two corresponding samples being P:S:C 40% and P:S:C 60%. The samples were examined via DLS to determine the size of the resulting nanocarriers. Figure 8 contains size distribution curves obtained from DLS measurements on pure block copolymer (blue), P:S (red), and P:S:C (black) solutions. Evident in both cases is an increase in the corresponding peak denoting populations in the range of ~100 nm, indicating aggregates with a larger R_h_ likely attributed to successful curcumin loading. Concurrently, an increase in the scattered intensity for the aggregate populations in the ~100 nm range is observed. Table 3 contains the R_h_ values at the peaks from DLS measurements one day and fourteen days after preparation of P:S:C nanocarriers. In both cases an increase in the R_h_ of larger aggregates is observed, as well as for the smaller aggregates in the P:S:C 40% sample, meaning that secondary aggregation occurs as time goes by.

**Figure 9:**
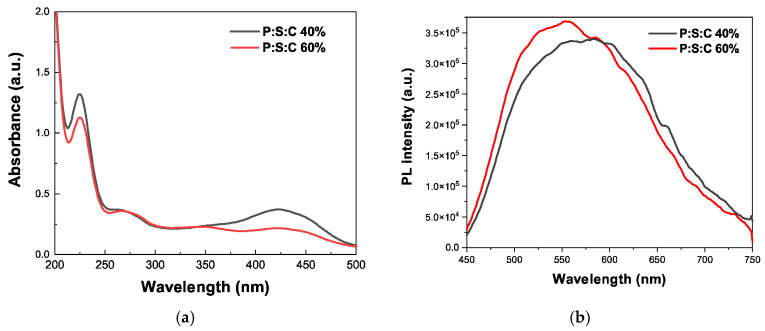
Results for P:S:C 40% and 60% CUR-loaded complexes from (**a**) UV–Vis measurements and (**b**) FS measurements.

UV–Vis and fluorescence spectroscopy measurements were employed to confirm the photophysical properties and the encapsulation efficiency and loading as well as the overall photophysical properties of the CUR-loaded structures. The corresponding results are shown in Table 3. The maximum encapsulation efficiency as calculated by UV–Vis using a curcumin calibration curve was observed for P:S:C 40% at 39% CUR. The characteristic absorption of curcumin at 420 nm and the slight shift in the wavelength indicates the success of the encapsulation [44]. More specifically, in Figure 9 the absorption at 424 nm for P:S:C 40% and at 421 nm for P:S:C 60% due to changes in the environment around the drug implies the loading of curcumin within the complexes. In Figure 9b, the maximum fluorescence emission peak of P:S:C 40% appears at 583 nm and at 557 nm for P:S:C 60%. The enhanced emission intensities compared to curcumin and surfactant in ethanol can be attributed to the aggregation-induced emission (AIE) phenomenon. The results of curcumin and surfactant in ethanol appear in Figure S8b and are in accordance with the literature [39].

**Figure 10:**
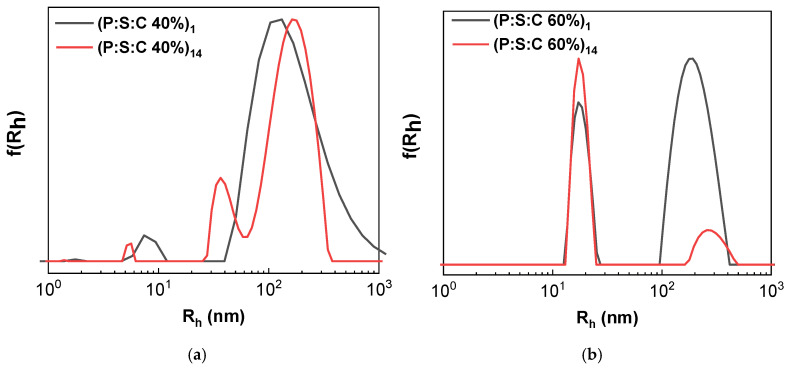
Size distributions from DLS measurements: (**a**) P:S:C 40% 14 days after preparation (red) compared to P:S:C 40% 1 day after preparation (black); (**b**) P:S:C 60% 14 days after preparation (red) compared to P:S:C 60% 1 day after preparation (black).

Stability investigation was conducted for the P:S:C formulations as well, via DLS measurements 14 days after the initial preparation. Corresponding measurements appear in Figure 10. A small shift in the peak center is observed in Figure 10a corresponding to an increased R_h_ due to aggregation of existing nanocarriers. On the other hand, in Figure 10b the peak centered at R_h_ = 18 nm showed a small increase in intensity while maintaining the same value for R_h_, whereas the peak initially centered at R_h_ = 193 nm had its center moved to R_h_ = 279 nm while greatly reducing in intensity. This indicates an increase in large nanocarrier size similar to P:S:C 40% along with a reduction in nanocarrier population and the formation of smaller aggregates. Stability was also examined through UV–Vis where the maximum absorption remained stable as depicted in Figure S9a. The FS measurements 14 days after preparation, shown in Figure S9b, indicate significantly lower emission in both P:S:C 40% and 60%. Some structural rearrangements have probably taken place within the complexes.

#### 3.2.2. Addition of NaCl to Curcumin-Loaded Nanocarriers

**Figure 11:**
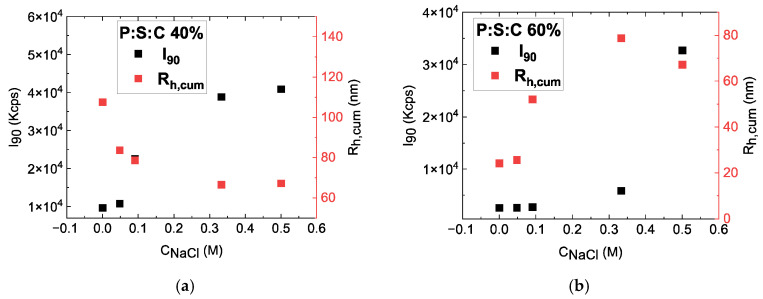
Intensity and R_h_ as a function of NaCl concentration for (**a**) P:S:C 40% and (**b**) P:S:C 60% formulations.

Since the systems under study pertain to nanostructures designed for potential biological applications, an important step is to examine the influence of NaCl concentration (i.e., solution salinity) on the obtained CUR-loaded complexes. Figure 11a shows that in P:S:C 40%, the R_h_ decreases with the increase in salt concentration, indicating that the particles fragment. Figure 11b shows the opposite behavior, meaning that NaCl favors further aggregation of drug-loaded complexes, as the increase in scattered intensity also indicates. However, significant changes in scattered intensity happen after 0.1 M and 0.3 M salinity, respectively, indicating that at least P:S:C 60% formulation is rather stable and well above the normal ionic strength of blood steam and leaving tissues.

#### 3.2.3. Interaction of Curcumin-Loaded Complexes with FBS

**Figure 12:**
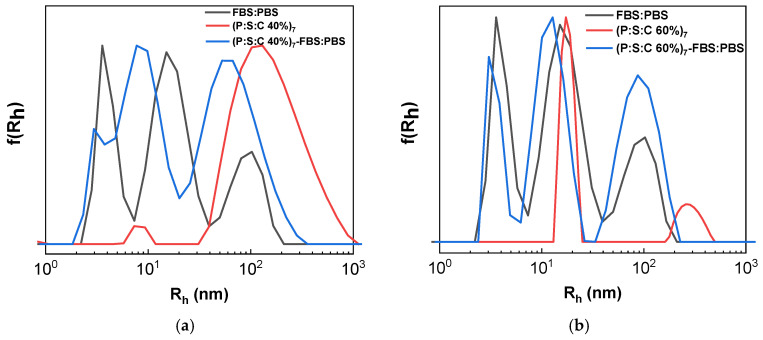
Results from DLS measurements: (**a**) P:S:C 40% on the 7th day after preparation with FBS:PBS (blue) compared to P:S:C 40% of the same day (red) and FBS medium (black); (**b**) P:S:C 60% on the 7th day after preparation with FBS:PBS (blue) compared to P:S:C 40% of the same day (red) and FBS medium (black).

The polyelectrolyte complexes containing curcumin were also examined in FBS:PBS solution as a crucial step for testing the properties of the nanocarriers in an environment that resembles the blood stream. For the investigation, FBS:PBS was initially measured in DLS in order to confirm the peaks produced by the medium [45] due to the presence of serum proteins (and their aggregates). The FBS peaks were present within all results when P:S:C was added in FBS:PBS. DLS results for P:S:C 40% in Figure 12a contain shifted peaks that allude to larger particles. The increase indicates the creation of small aggregates due to interaction with proteins. Sample P:S:C 60% in protein medium shows no significant alteration (Figure 12b), showing no changes in the aggregation behavior of nanocarriers due to interaction with the medium. The results confirm the possible applicability of the nanocarriers for further investigation for biomedical applications.

#### 3.2.4. Cryo-TEM Imaging of P:S:C 40% and P:S:C 60% Formulations

**Figure 13:**
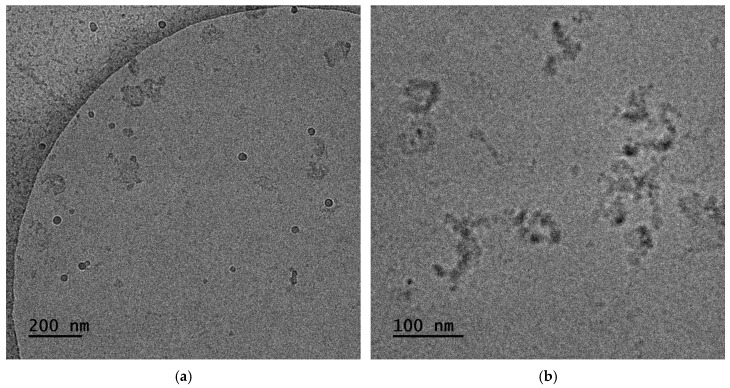
Results from cryo-TEM for (**a**) P:S:C 40% and (**b**) P:S:C 60% formulations.

Cryo-TEM analysis was also conducted for the P:S:C formulations obtained in this study. With the cosolvent protocol, nanocarriers P:S:C are created—consisting of P:S and encapsulated curcumin. The particles observed are spherical or worm-like and show clustering, where the darker shaded regions are supposed to have a higher density relative to other regions of the particle, indicating curcumin encapsulation. The appearance of non-spherical particles in the sample stems from the creation of randomly aggregated mixed micelles during the initial stages of PEC formation (Figure 13a). Similar to this is the case of P:S:C 60%, which is shown in Figure 13b, where again curcumin encapsulation seems to be achieved in the corresponding particles. In P:S:C 60% the aggregation of primary complexes creates more worm-like particles and to a greater extent.

## 4. Conclusions

The formation of nanocarriers by a neutral–cationic POEGMA-b-Q(PDMAEMA) double hydrophilic block copolymer combined with the anionic surfactant sodium oleate was reported. The structure and physicochemical properties of the complexes formed by electrostatic interactions depend on the NaOL content in the formulation. The efficient encapsulation of curcumin inside the complexes was demonstrated via cryo-TEM, DLS, UV–Vis, and FS measurements. The cryo-TEM revealed clusters of vesicles and worm-like micelles, with the darker shaded areas of the particles signifying efficient curcumin encapsulation. DLS measurements denoted populations around 100 nm, assuming aggregates with greater R_h_ and hence successful curcumin loading. The maximum encapsulation efficiency of curcumin was 39% and the maximum encapsulation loading was 2.63% for the P:S:C 40% sample. Due to curcumin’s characteristic absorption at 420 nm, curcumin loading was evaluated by UV–Vis experiments. The contribution of FS measurements lies in the evidence of AIE phenomenon. Stability of loaded nanoparticles was ascertained via DLS measurements two weeks after preparation of the samples. Furthermore, the impact of increasing concentration of NaCl and FBS to nanoparticle size indicated suitability of those complexes for biological applications. A possible route for future experiments is a detailed look at the impact of various drug molecules and surfactants on PEC morphology and stability. Finally, another research direction is the elucidation of drug release kinetics from PEC-based nanocarriers.

## Figures and Tables

**Table 1:** Results from DLS, FS, and ELS measurements for the block copolymer, sodium oleate, and complexes at different P:S ratios.

Sample	I_90_ (Kcps)	PDI	R_h,cont_ (nm)	Zeta Potential (mV)	I_1_/I_3_
P	430	0.27	8.5 84	42.2	1.83
S	48,800	0.29	39 172	−71	0.93
P:S 20%	1015	0.42	16 84	18	1.32
P:S 40%	1128	0.20	20 110	−0.15	1.19
P:S 60%	1836	0.42	19 139	−30	1.14
P:S 80%	4050	0.47	11 34 160	−32	1.14

**Table 2:** Results of the stability tests, one week after preparation, by DLS experiments of the four different complexes prepared.

Sample	I_90_ (Kcps)	PDI	R_h,cont_ (nm)
P:S 20%	426	0.44	15 89
P:S 40%	364	0.38	36
P:S 60%	511	0.32	19 64
P:S 80%	412	0.47	19 64 343

**Table 3:** DLS results for complexes and CUR-loading calculations for P:S:C 40% and P:S:C 60% formulations.

Sample	Curcumin Maximum Loading (wt%)	R_h,cont_ (nm) Day 1	R_h,cont_ (nm) Day 14	Encapsulation Efficiency %	Loading %
P:S:C 40%	5	8 156	37 163	39	2.63
P:S:C 60%	5	18 193	18 279	24	1.39

## Data Availability

The original contributions presented in this study are included in the article/Supplementary Materials. Further inquiries can be directed to the corresponding author.

## Supplementary Materials

The following supporting information can be downloaded at https://www.mdpi.com/article/10.3390/ma18235375/s1, Figure S1. Size distribution from DLS for (POEGMA-b-Q(PDMAEMA) copolymer aqueous solution (C = 1 × 10^−3^ g/mL); Figure S2. Size distribution from DLS for sodium oleate aqueous solution (C = 1 × 10^−3^ g/mL); Figure S3. Plots of (a) scattered intensity, (b) R_h,cum_, (c) zeta potential, (d) PDI vs. the concentration of sodium oleate; Figure S4. Calibration curve of curcumin in ethanol; Figure S5. (a) UV—Vis of sodium oleate/curcumin solution in aqueous medium, (b) FS spectrum of sodium oleate/curcumin solution in aqueous medium; Figure S6. (a) UV—Vis of sodium oleate/curcumin solution in ethanol, (b) FS spectrum of sodium oleate/curcumin solution in ethanol; Figure S7. Picture of curcumin in ethanol (left) and sodium oleate/curcumin/ethanol solution under UV lamp (λex = 365 nm); Figure S8. (a) UV—Vis spectra from stability studies of P:S:C 40% one day after preparation (black) and two weeks after preparation (red), (b) FS spectra from stability studies of P:S:C 40% one day after preparation (black) and two weeks after preparation (red); Figure S9. (a) UV—Vis spectra from stability studies of P:S:C 60% one day after preparation (black) and two weeks after preparation (red), (b) FS spectra from stability studies of P:S:C 60% one day after preparation (black) and two weeks after preparation (red).
