# Supplementary material for: Block Copolymer–Sodium Oleate Complexes Through Electrostatic Interactions for Curcumin Encapsulation
Source: Materials (Basel). 2025 Nov 28;18(23):5375. doi: 10.3390/ma18235375 (PMC12693471; doi:10.3390/ma18235375)
Supplement: Supplementary file 1 [file materials-18-05375-s001.zip › materials-3960519-supplementary.pdf]

# Block Copolymer–Sodium Oleate Complexes Through Electrostatic Interactions for Curcumin Encapsulation

**Evanthia Ganou[1,2], Michaila Akathi Pantelaiou[1,2], Varvara Chrysostomou[1], Karolina Olszowska[3], Barbara Trzebicka[3] and Stergios Pispas[1,*]**

[1] Theoretical and Physical Chemistry Institute, National Hellenic Research Foundation, 48 Vassileos Constantinou Ave., 11635 Athens, Greece; euaganou@gmail.com (E.G.); akathi39@gmail.com (M.A.P.); chrysostomou.v@gmail.com (V.C.)

[2] Department of Chemistry, National and Kapodistrian University of Athens, Panepistimiopolis Zografou, 15771 Athens, Greece

[3] Centre of Polymer and Carbon Materials, Polish Academy of Sciences, 34 ul. M. Curie-Skłodowskiej, 41-819 Zabrze, Poland; kolszowska@cmpw-pan.pl (K.O.); btrzebicka@cmpw-pan.pl (B.T.)

* Correspondence: pispas@eie.gr

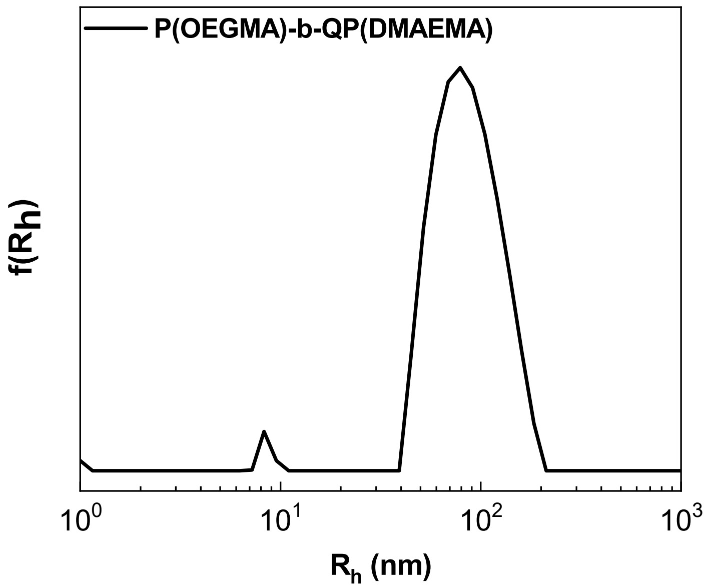


**Figure S1**. Size distribution from DLS for (POEGMA-b-Q(PDMAEMA) copolymer aqueous solution (C=1*$10^{-3}$ g/mL)

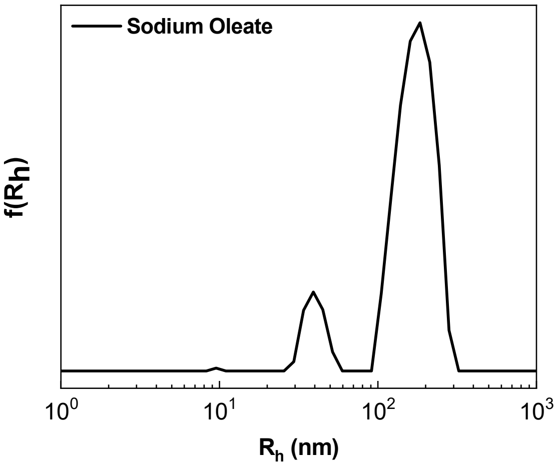


**Figure S2.** Size distribution from DLS for sodium oleate aqueous solution (C= $1\times10^{-3}$ g/mL)

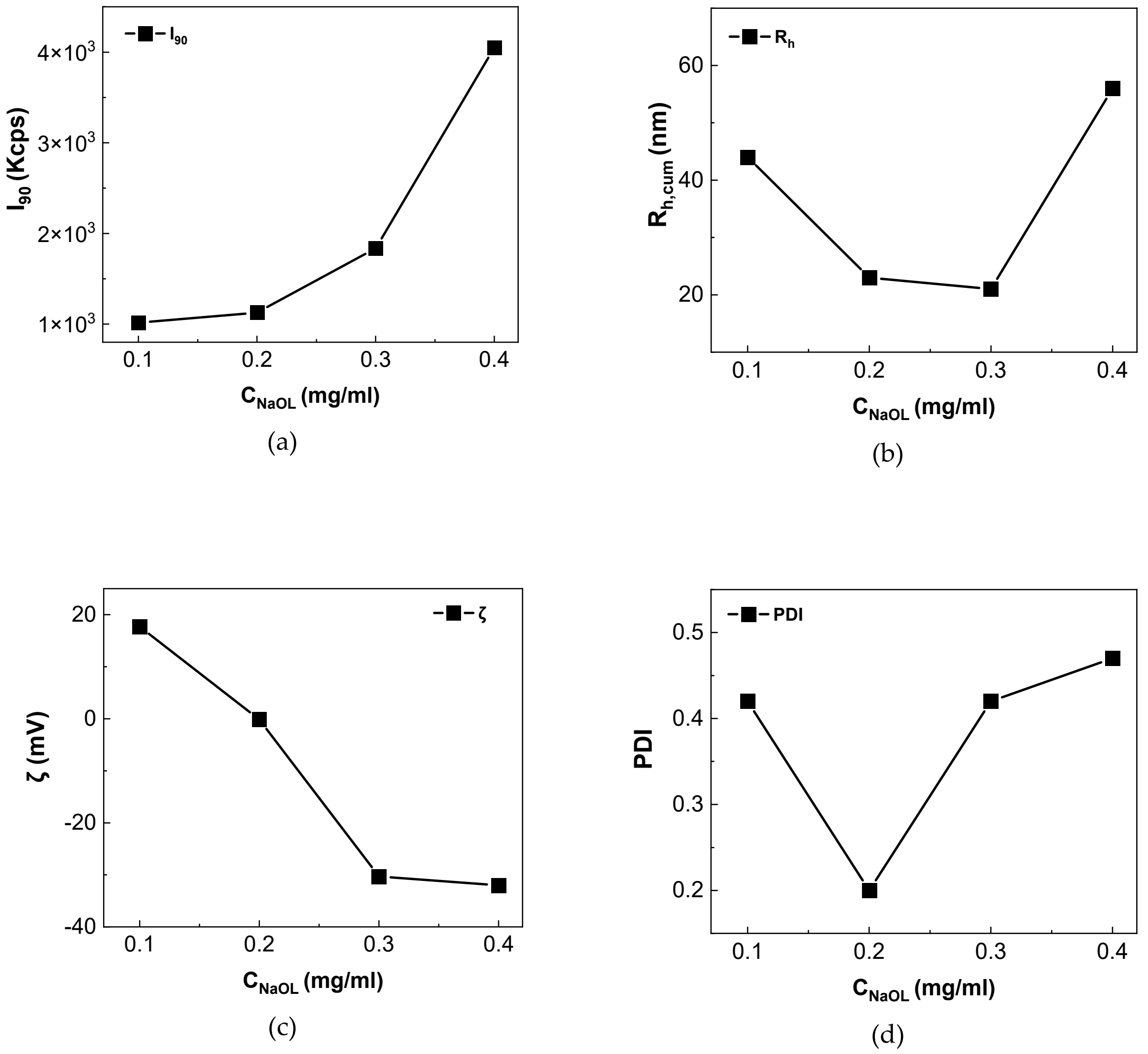


**Figure S3.** Plots of (a) scattered intensity; (b) $R_{h,cum}$; (c) zeta potential; (d) PDI index vs. the concentration of sodium oleate

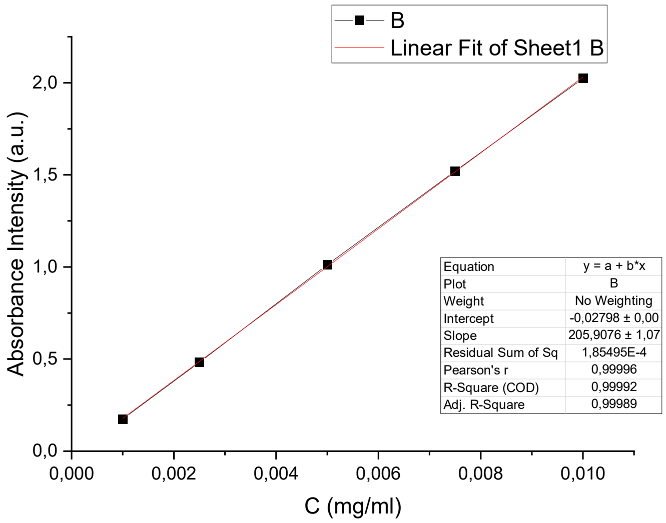


| C ( mg/ml) | Absorbance Intensity (a.u.) |
|---|---|
| 0.01 | 2.02 |
| 0.0075 | 1.52 |
| 0.005 | 1.01 |
| 0.0025 | 0.48 |
| 0.001 | 0.17 |

**Figure S4.** Calibration Curve of curcumin in ethanol

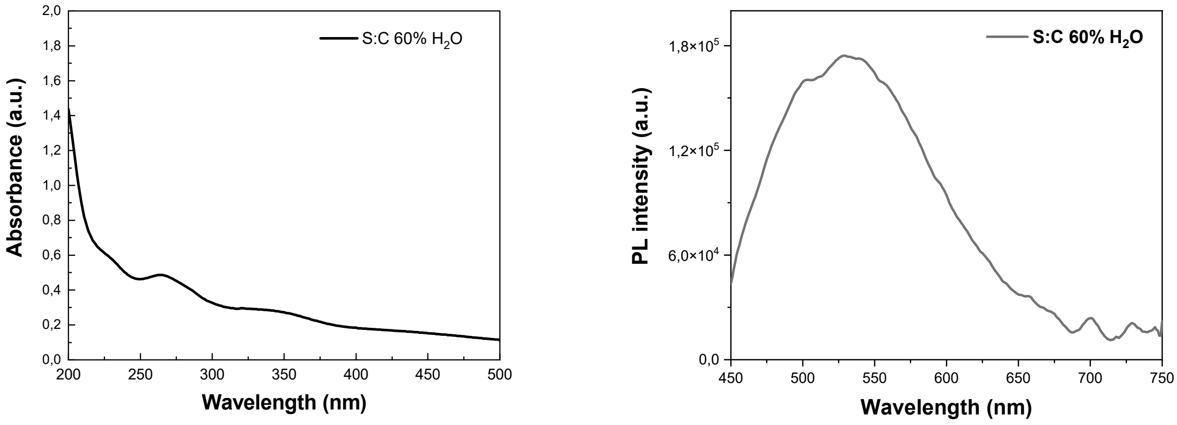


(a) (b)

**Figure S5.** (a) UV-Vis of sodium oleate/curcumin solution in aqueous medium; (b) FS spectrum of sodium oleate/curcumin solution in aqueous medium

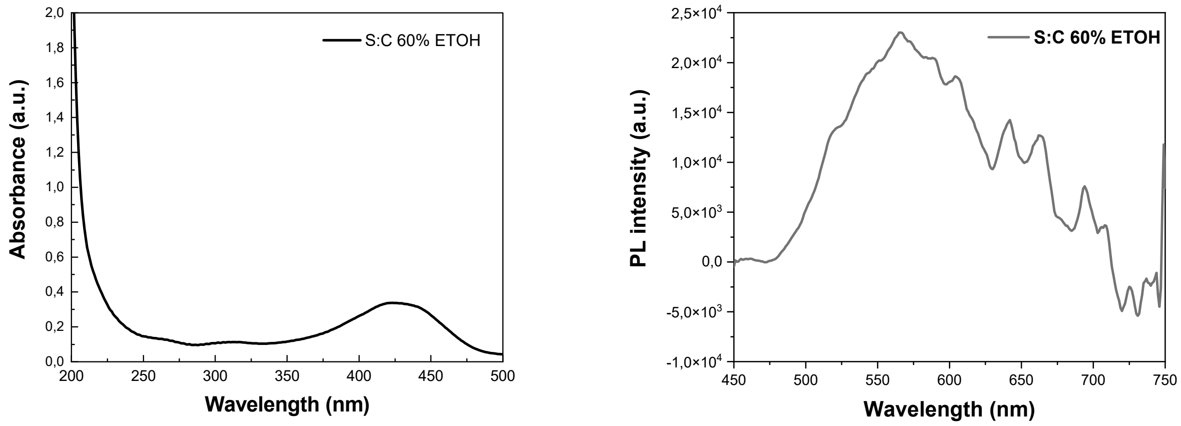


(a) (b)

**Figure S6.** (a) UV-Vis of sodium oleate/curcumin solution in ethanol; (b) FS spectrum of sodium oleate/curcumin solution in ethanol

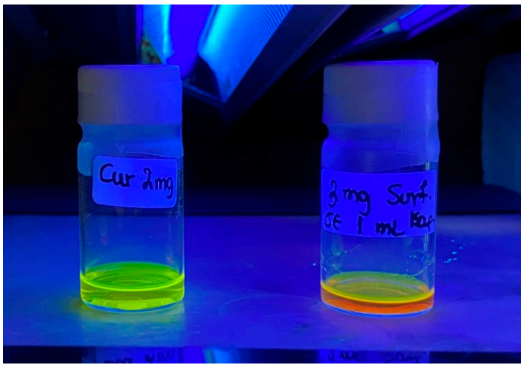


**Figure S7.** Picture of curcumin in ethanol (left) and sodium oleate/curcumin-ethanol solution under UV lamp ($\lambda_{ex}$=365 nm)

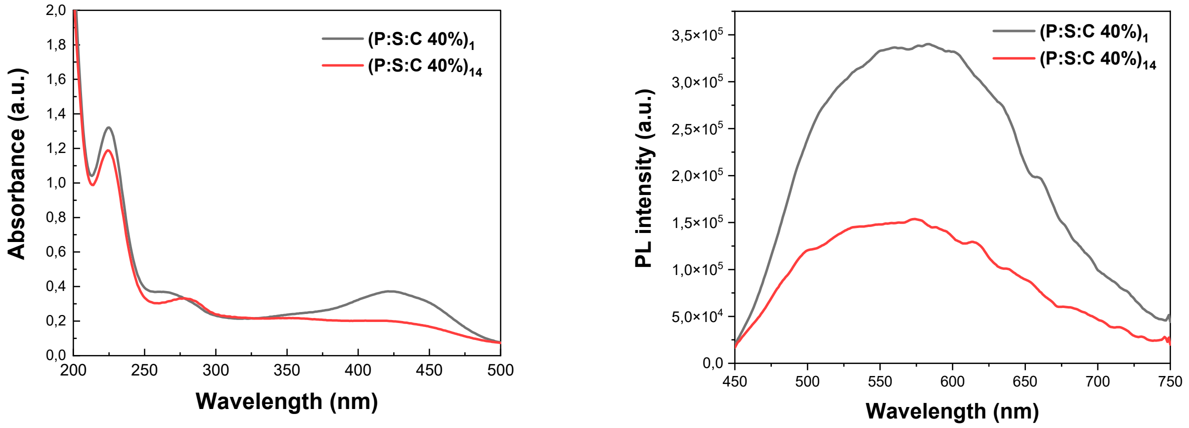


(a) (b)

**Figure S8.** (a) UV-Vis spectra from stability studies of P:S:C 40% one day after preparation (black) and two weeks after preparation (red); (b) FS spectra from stability studies of P:S:C 40% one day after preparation (black) and two weeks after preparation (red)

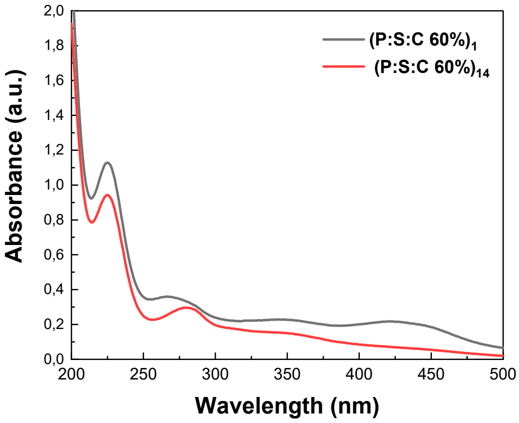


(a)

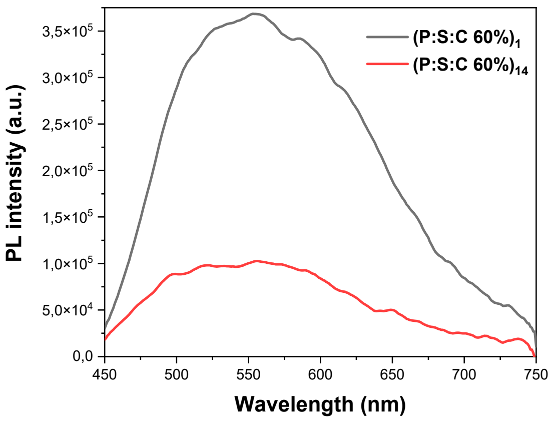


(b)

**Figure S9.** (a) UV-Vis spectra from stability studies of P:S:C 60% one day after preparation (black) and two weeks after preparation (red); (b) FS spectra from stability studies of P:S:C 60% one day after preparation (black) and two weeks after preparation (red)
